# RNA-stabilization factors in chloroplasts of vascular plants

**DOI:** 10.1042/EBC20170061

**Published:** 2018-02-16

**Authors:** Nikolay Manavski, Lisa-Marie Schmid, Jörg Meurer

**Affiliations:** Plant Sciences, Faculty of Biology, Ludwig-Maximilians-University Munich, Großhaderner Street 2-4, 82152, Planegg-Martinsried, Germany

**Keywords:** chloroplast gene expression, plastid RNA metabolism, RNA binding proteins, RNA stability, RNA processing, RNases

## Abstract

In contrast to the cyanobacterial ancestor, chloroplast gene expression is predominantly governed on the post-transcriptional level such as modifications of the RNA sequence, decay rates, exo- and endonucleolytic processing as well as translational events. The concerted function of numerous chloroplast RNA-binding proteins plays a fundamental and often essential role in all these processes but our understanding of their impact in regulation of RNA degradation is only at the beginning. Moreover, metabolic processes and post-translational modifications are thought to affect the function of RNA protectors. These protectors contain a variety of different RNA-recognition motifs, which often appear as multiple repeats. They are required for normal plant growth and development as well as diverse stress responses and acclimation processes. Interestingly, most of the protectors are plant specific which reflects a fast-evolving RNA metabolism in chloroplasts congruent with the diverging RNA targets. Here, we mainly focused on the characteristics of known chloroplast RNA-binding proteins that protect exonuclease-sensitive sites in chloroplasts of vascular plants.

## Introduction

The plastid is the result of an endosymbiotic event that occurred billion years ago and comprised the incorporation of a cyanobacterium into a eukaryotic, mitochondria-possessing cell. The main benefit of the cyanobacterium for the host cell was the supply of additional energy and photoautotrophic growth, granting an enormous selective advantage. The genome of the endosymbiont underwent dramatic transformations, whereby the majority of the cyanobacterial genes were transferred into the nucleus or lost [1]. Meanwhile, about half of the plastid proteome consists of members, which are not of cyanobacterial origin. The genome of the model plant *Arabidopsis thaliana* contains a total of 87 genes encoding mostly ribosomal and photosynthetic subunits, 4 rRNA and 37 tRNA genes [2–4].

Similar to the operons of the cyanobacterial ancestor, the chloroplast genes are primarily organized in newly composed transcriptional units and often contain genes that do not belong to the same functional cluster. These units are transcribed as polycistronic pre-mRNAs by two types of RNA polymerases: the plastid-encoded bacterial type RNA polymerase (PEP) and at least one phage-type nuclear-encoded RNA polymerase (NEP). The PEP is composed of four basic plastid-encoded core subunits (*rpo* genes) and requires additional nucleus-encoded sigma factors (six in *Arabidopsis*) for promoter recognition and transcription initiation as well as further factors, which associate to the core during chloroplast development. In contrast, NEP is a one-subunit-enzyme which seems to require additional accessory factors as well. It was assumed that photosynthetic genes are predominantly transcribed by the PEP, while genes for the translational machinery and for PEP subunits are targets of the NEP [5]. However, most plastid genes contain promoter elements that are recognized by both polymerases. NEP and PEP promoters are not restricted to the transcriptional units, but are also found in intergenic regions and opposite to annotated genes, where they are responsible for the synthesis of the recently identified chloroplast noncoding RNAs [6], whose role is poorly understood. In contrast with cyanobacteria, the transcriptional regulation in plastids is global and large-scale rather than gene-specific. Both polymerase types are present and active during all stages of chloroplast development as well as in nongreen tissues. Upon light exposure and formation of the photosynthetic thylakoid membrane both, PEP and NEP, exhibit highest activity and remain active during the entire development of the leaf while in mature chloroplasts genes are preferentially transcribed by the PEP [7]. In most mutants generally affected in the expression of plastid genes including *rpo* genes and genes for the translational machinery, transcripts preferentially generated by the NEP predominate [8–10].

Even though there are examples for gene-specific transcriptional regulation, such as the *psbD*–*psbC* promoter switch in response to blue light in barley, gene-specific transcriptional regulation seems to be an exception rather than the rule [11,12]. Unlike in bacteria, little is known about processes of plastid transcription termination, which seems to be assisted by additional gene-specific factors [13]. Most obviously, the most pronounced difference between cyanobacteria and chloroplasts in terms of regulation of gene expression is the level of control. In bacteria, gene expression is mainly regulated via transcription initiation, while in chloroplasts post-transcriptional control predominates. The chloroplast retained some of the ancestral attributes such as a similar translational machinery, the operon-like gene organization and bacteria-related RNA degradation processes. However, over the course of evolution the chloroplast recruited many new features including the acquisition of introns, the ability to post-transcriptionally modify the RNA sequence, a process called RNA editing, and extensive processing events of the polycistronic pre-mRNAs leading to complex transcript patterns [14]. These new features together with the coevolution of numerous mostly newly acquired, nucleus-encoded RNA-binding proteins involved in the expression of chloroplast genes opened up new possibilities for targeted and more precise regulation of gene products at many different steps that enable adaptive and developmentally flexible chloroplast biogenesis and plant viability [15]. The processing of plastid polycistronic transcripts into complex mRNA isoforms is one of the most obvious differences between bacteria and chloroplasts and offers a platform for gene-specific regulation through RNA lifetime and translational control [14,16–18].

The conversion of polycistronic transcripts into oligocistronic or monocistronic isoforms, the post-transcriptional generation of 5′ and 3′ transcript termini as well as RNA decay in general is accomplished by plastid ribonucleases [19]. In chloroplasts, like in bacteria, two types of ribonucleases exist, endonucleases that execute internal RNA cleavage, and exonucleases that progressively remove nucleotides from the end of the RNA in the either 5′→3′ or 3′→5′ direction. In *Arabidopsis*, 17 gene products assigned to exhibit ribonucleolytic activity or to contain domains characteristic for ribonucleases were predicted to be localized in the chloroplast [19]. Among the endonucleases, the RNase J and RNase E are thought to be the most relevant enzymes for intercistronic mRNA cleavage in chloroplasts [16,17], while RNase Z (TRZ2) and PRORP are important for the maturation of tRNAs [19]. CSP41(a/b) have been shown to exhibit endonucleolytic activity and may serve to stabilize a large set of nontranslated target mRNAs and precursor rRNAs during the night [17,20,21]. Two mini-RNase III proteins (RNC3 and RNC4) have been demonstrated to be important for rRNA maturation and intron recycling [22]. Three exonucleases have been reported to be active in *Arabidopsis* chloroplasts, RNR1 (RNase R) and PNPase with 3′→5′ specificity, and RNase J with 5′→3′ activity. As in other organisms, plant ribonucleases are thought to be rather unspecific and certain substrate preferences might be determined by the RNA structure or chemical features and less by the RNA sequence [17,19].

Based on the current knowledge, it has been proposed that both RNA decay and intercistronic processing of polycistronic units are initiated by housekeeping endonucleases (RNase E and J, CSP41) that cleave the RNA in unstructured, unprotected regions [16]. Although accessibility of the RNA target is hypothesized to be determined by the extent of the association with RNA-binding proteins or ribosomes, the precise parameters for initiating the endonucleolytic attack and a regulatory impact of this activity remain unknown [16,19]. In a second step, the endonucleolytically cleaved RNA products are either stabilized to form translationally competent RNAs or rapidly degraded by the PNPase stimulated by polyadenylation that is accomplished either by the PNPase itself or by another yet unidentified plastid poly(A) polymerase [23]. The activity of the PNPase is impeded by secondary structures that are often found in the 3′-untranslated region of mRNAs. These structures are formed by short inverted repeat sequences (IP) that are also present in bacterial RNAs where they perform a different function, namely the termination of transcription. Thus, one of the parameters that determines RNA lifetime are stable secondary structures that could be found in several plastid mature RNA 3′ ends. The transcript 5′ ends are thought to be predominantly protected by tightly bound RNA-binding proteins, which also determine the mature 5′ transcript termini by serving as barriers for 5′→3′ exonucleases. In addition, protecting stem–loop structures also found in chloroplast transcript 5′ ends can be relevant for regulation of mRNA stability and translation as they might hinder ribosomal binding, i.e. access to the Shine–Dalgarno sequence. RNA-binding proteins also protect and define the 3′ termini in transcripts lacking prominent inverted repeat sequences [24]. It seems that the lifetime of chloroplast transcripts is mainly governed by protective RNA-binding proteins as well as by the structure of the RNA and presumably to a certain extent the regulation and/or expression of unspecific RNases.

Most of the plastid RNA protectors present in vascular plants are not found in green algae, such as Chlamydomonas, and vice versa, reflecting the diverged genome–plastome interaction and the tight coevolution of the cellular genetic compartments in the respective lineages during endosymbiosis. Comparably dissimilar are post-transcriptional processes in plastids of land plants and green algae. For example, RNA editing requires a plethora of trans-acting factors and is widespread in vascular plants but entirely absent in green algae [25]. Often, chloroplast RNA-binding proteins required for different post-transcriptional processing steps were found to form either homomultimeric or heteromeric proteinaceous and/or RNA-containing high molecular complexes mostly of unknown composition (e.g. [10,13,26–32]).

**Figure 1:**
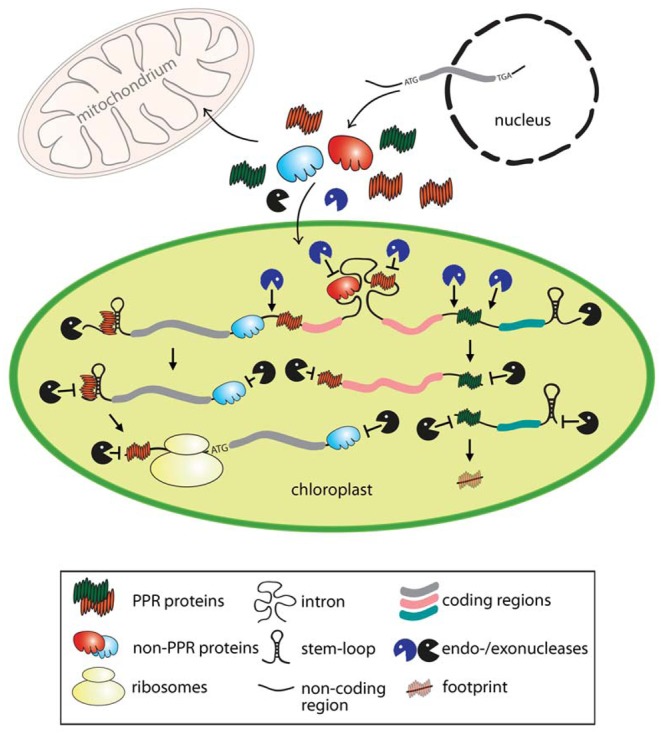
Model for the function of nuclear-encoded PPR and other RNA-binding proteins in protecting plastid RNAs from ribonucleolytic attack The mode of action shown is representative also for mitochondria. The two colors of protectors (PPR and non-PPR proteins) point to the specificity for particular targets. The scheme shows the endo- and exonucleolytic processing of a representative tricistronic precursor transcript resulting in three stable and translational competent monocistronic products. In chloroplasts, unprotected sites of precursor mRNAs are cleaved by site-specific endonucleases (blue) often in intergenic regions, giving rise to new 5′ and 3′ transcript ends. These extremities are protected against attacks of exonucleases (black) either by the specific binding of RNA protectors and/or by stabilizing secondary RNA stem–loop structures at both ends. For example, PPR10, HCF152, and CRP1 delineated in green show that binding to intergenic regions and subsequent endonucleolytic processing of both adjacent regions results in RNAs with overlapping 5′- and 3′-UTRs, which are protected by the same PPR protein. Binding of protectors to endonuclease-sensitive sites (e.g. intron regions) prevents the cleavage and subsequent unspecific exonucleolytic degradation of the processed RNAs. Furthermore, binding of these factors can stimulate the restructuring of the 5′ region that in turn promotes translation initiation. Short noncoding RNA fragments resulting from the protective role of tightly bound PPR proteins are shown as footprints.

In this review, we provide an overview about the protective role of mostly vascular plant-specific RNA-binding proteins in chloroplasts and highlight the progress made over two decades. We outline 18 known nucleus-encoded RNA-stabilizing factors according to the protein class they belong to: (1) pentatricopeptide repeat (PPR) proteins, (2) chloroplast ribonucleoproteins (cpRNPs), and 3) additional RNA protectors, which do not belong to a larger protein family. Our review article mainly focuses on vascular plant chloroplasts as gene expression in *Chlamydomonas reinhardtii* differs in many respects from vascular plants and extensive reports deserve an independent comprehensive review article. The reader is referred to recent articles reporting on plastid mRNA stability in this unicellular algae (e.g. [17,33–41] ). An overview of the described plastid factors, including their preferential targets and classification of their domains is given in Table 1. Figure 1 summarizes the main mechanisms how binding of RNA protectors confers transcript stability in chloroplasts.

**Table 1:** List of known chloroplast RNA protectors, their targets, and domains

Protein	Gene ID	Targets/Binding Site(s)	Protein Family/Domains	Organism	References
**PPRs**					
PPR10	GRMZM2G177169	*atpH* 5′ UTR, *psaJ* 3′ UTR	PPR	*Zea mays*	[24,52]
HCF152	AT3G09650	*psbH-petB* IGR, *petB* 5′ UTR, *psbH* 3′ UTR	PPR	*Arabidopsis thaliana*	[57,28]
CRP1	GRMZM2G083950	*psaC* 5′ UTR, *petA* 5′ UTR (role in translation)	PPR	*Zea mays*	[26,60,62]
	AT5G42310	*petB-petD* IGR (shown in *Arabidopsis*)		*Arabidopsis thaliana*	
SOT1	AT5G46580	*rrn23* 5′ end	PPR-SMR	*Arabidopsis thaliana*	[63–65]
PPR53	GRMZM2G438524			*Zea mays*	
PGR3	AT4G31850	*petL, ndhA* 5′ UTRs	PPR	*Arabidopsis thaliana*	[66–69]
	GRMZM2G372632			*Zea mays*	
PPR5	GRMZM2G025409	*trnG(UCC)* intron	PPR	*Zea mays*	[61,70]
	AT4G39620			*Arabidopsis thaliana*	
EMB175	AT5G03800	*rpl16* 5′ UTR	PPR-PLS-DYW	*Arabidopsis thaliana*	[72,73]
PPR103	GRMZM2G170896			*Zea mays*	
SVR7	AT4G16390	*atpF-atpA* IGR, *psaJ-rpl33* IGR, *atpB/E* 5′ UTR	PPR	*Arabidopsis thaliana*	[75,76]
ATP4	GRMZM2G128665			*Zea mays*	
CRR2	At3g46790	*rps7-ndhB* IGR	PPR-PLS-DYW	*Arabidopsis thaliana*	[77,78]
PpPPR_38	BAF02672.1	*clpP-rps12* IGR	PPR	*Physcomitrella patens*	[79,80]
**cpRNPs**					
28RNP	X57955.1	*psbA, rbcL, petD, rps14* 3′ UTRs	cpRNPs, RRM motif	*Spinacea oleracea*	[85]
CP31A	At4G24770	3′ ends of *psbB, psbD, psaA/B, atpB, ndhB, ndhF*	cpRNPs, RRM motif	*Arabidopsis thaliana*	[88,89]
CP29A	AT3G53460	3′ ends of *psbB, psbD, psaA/B, atpB, ndhB*	cpRNPs, RRM motif	*Arabidopsis thaliana*	[98]
CP33A	AT3G52380	Multiple transcripts	cpRNPs, RRM motif	*Arabidopsis thaliana*	[90]
**Additional chloroplast RNA-stabilizing RNA-binding proteins**					
HCF145	AT5G08720	*psaA* 5′ UTR	2 TMR domains and 2 SRPBCC domains	*Arabidopsis thaliana*	[91,92]
	XM_001783694			*Physcomitrella patens*	
PrfB1/HCF109	AT5G36170	Transcripts with UGA stop codons	ribosomal release factor, SPF and GGQ motifs	*Arabidopsis thaliana*	[94,95]
PrfB3	AT3G57190	*petB* 3′ UTR	homologous to PrfB1 (HCF109)	*Arabidopsis thaliana*	[31]
HCF107	AT3G17040	*psbH* 5′ UTR	HAT (Half a TPR)	*Arabidopsis thaliana*	[29,56,97–99]
Zm-HCF107	GRMZM2G121960			*Zea ma*ys	

Abbreviation: IGR, intergenic region. Homologs are shown in the same row.

## Specificity factors for plastid RNA stabilization

### Pentatricopeptide repeat proteins

The family of plant PPR proteins presumably evolved from tetratricopeptide repeat (TPR) proteins and consists of more than 450 up to approximately 1000 conserved members, shared among the eukaryotic kingdom [42,43]. PPR proteins are characterized by their modular and superhelical structure, consisting of 2 to approximately 30 PPR motifs ranging in length from 31 to 36 amino acids [43,44], which were shown to be essential for sequence-specific one-repeat:one-nucleotide RNA recognition including combinatorial effects of two amino acids in each repeat [45,46]. PPR proteins fulfill essential functions in both, chloroplast and mitochondrial RNA metabolism, including endonucleolytic processing, splicing, editing, and translation initiation [44,47]. A subset of PPR proteins in both plant organelles was shown to be particularly important for the stabilization of mRNAs, rRNAs, and tRNAs [44]. The underlying mechanisms involve specific binding of the PPR proteins to the 5′-UTR, 3′-UTR, or other exo-/endonuclease-sensitive sites of certain transcripts that block their degradation by ribonucleases [48]. In contrast with the predominant PPR proteins in plant organelles, the 38–40 amino acid degenerate repeats of the octatricopeptide repeat (OPR) family expanded in Chlamydomonas [38,39,49]. According to mutant phenotypes and the architectural similarities to PPRs, OPRs are assumed to form α-helical RNA-binding domains and are implicated in a variety of post-transcriptional steps. This model of action is further supported by the discovery of short RNA footprints that accumulate as sRNAs, caused by association of RNA-binding proteins to their targets in monocots, dicots, and Chlamydomonas [24,48,50,51]. Several of these PPR proteins were reported to not only stabilize a certain transcript but also hold an additional function in stimulating its editing, splicing, and/or translation.

#### PPR10 protects the 5′ and 3′ ends of *atpH* and *psaJ* respectively, and facilitates the translation of *atpH* mRNA

The best understood PPR protein is PPR10 in maize. PPR10 is essential for the stabilization of two different RNA termini: the 5′-UTR of *atpH* and *rpl33* as well as the 3′-UTR of *atpI* and *psaJ*, whose intergenic regions (*atpI–aptH* and *psaJ–rpl33*) share consensus sequences to which PPR10 specifically binds [24] (Figure 1). In concert with exoribonucleases, PPR10 is able to define the 5′ and 3′ termini of processed transcripts which overlap in the intergenic region and contributes thereby to the maturation of their RNA termini [52]. Studies of the PPR10 crystal structure showed that it forms homodimers when bound to *psaJ*, offering new RNA-binding regulation perspectives via different dimerization states [53]. However, the solution structure as well as more detailed studies on the mode of RNA recognition came to the conclusion that PPR10 binds to its target RNAs in a monomeric form [45,54]. Finally, PPR10 was shown to enhance the translation of *atpH* by restructuring its 5′ region and exposing its otherwise masked ribosome-binding site [52]. A similar mechanism could also be shown for at least two other PPR proteins, which provide sequence specificity for other 5′ and 3′ transcript termini: HCF152 and CRP1.

#### HCF152 binds to the *psbH–petB* intergenic region and is involved in the processing and stabilization of *petB* transcripts

In a first step to identify novel factors in *Arabidopsis* involved in chloroplast gene expression and the establishment of the photosynthetic thylakoid membrane, a large number of *high chlorophyll fluorescence* (*hcf*) mutants were screened [55]. The *hcf152* mutant showed defects in the processing and accumulation of the *psbB–psbT–psbH–petB–petD* transcript cluster accompanied by severe defects in PSII and cytochrome *b*_6_*f* complex formation [27,56]. Further analysis revealed that the corresponding HCF152 protein forms an RNA-binding homodimer [57]. A screen for small noncoding RNAs in chloroplasts identified an abundant and conserved candidate in the *psbH–petB* intergenic region in monocots and dicots [48,50,58], supporting the preferential binding of HCF152 to this region, which also corresponded with the immediate 5′ or 3′ termini of *petB* and *psbH* RNAs respectively, as revealed by precise transcript mapping [24]. *In vitro* RNA-binding experiments validated that this sRNA represents the direct target of HCF152, suggesting that HCF152 protects the cleaved 5′ *petB* and 3′ *psbH* RNAs with identical sequences in their termini against exonucleolytic degradation, which resembles the function of PPR10 [48,24].

#### CRP1 stabilizes *petB* 3′ and *petD* 5′ ends and regulates translation of additional targets in maize and Arabidopsis

The maize mutant *crp1* (*chloroplast RNA processing 1*) was identified in a screen for mutants with distinct defects in the expression of plastid-encoded genes. It was shown to lack the cytochrome *b*_6_*f* complex, which could be traced back to defects in the accumulation of monocistronic *petB* and *petD* transcripts [59]. In addition, CRP1 was shown to be important for translation initiation of *petA, petD*, and *psaC* mRNAs [26]. *In vivo* RIP-chip experiments with the 5′-untranslated regions of *psaC* and *petA* mRNAs further contributed to the understanding of CRP1 function as a translational regulator [60]. Electrophoretic mobility shift assays with recombinant ZmCRP1 verified the preferential binding to the *petA* 5′-UTR [61]. Further analysis of the *Arabidopsis* homolog AtCRP1 revealed an association with the 5′-UTR of *psaC*, the *petB–petD* intergenic region and to a much weaker extent the *petA* 5′-UTR [62]. This is further supported by the identification of footprints in those three RNA targets [51]. Taken together, although the strength of binding to the targets differs between *Arabidopsis* and maize the overall analyses argue for similar functions of both CRP1 proteins in stabilization of *petD* 5′ and *petB* 3′ RNA termini and in translational regulation of *psaC* and *petA* mRNAs.

#### PPR53 (*Zea mays*) and SOT1 (*Arabidopsis thaliana*) stabilize the 5′ end of the 23S rRNA precursor and stimulate the translation of *ndhA*

Analysis of the *Zea mays* mutant *ppr53* revealed that 5′ transcript ends of *rrn23* and *ndhA* RNAs were absent. PPR53 was able to bind to the 5′ end of the *rrn23* RNA, which was also predicted to be the PPR53 target site, but failed to do so in case of the 5′ end of *ndhA* RNA. Thus, the authors concluded that PPR53 stabilizes the 23S rRNA by binding to its 5′ region and stimulates the translation of *ndhA* via another yet unknown factor(s) [63]. Experiments on the *Arabidopsis* ortholog SOT1 suggest that the binding of SOT1 to the 5′ end of the dicistronic 23S-4.5S rRNA prevents its exonucleolytic degradation [64]. Moreover, the SOT1 binding site was narrowed down to an RNA segment of 14 nucleotides and an endonuclease activity was attributed to its small MutS-related (SMR) domain, pointing to a role in stabilization and maturation of 23S-4.5S rRNA precursors [65].

#### Proton gradient regulation3 is responsible for the stability of *petL* and *ndhA* whose translation it activates

Proton gradient regulation3 (PGR3) is an example for another PPR protein that harbors two functions: transcript stabilization and translation initiation. Initial experiments including different *pgr3* alleles revealed defects in cytochrome *b*_6_*f* and NDH complex accumulation. These defects were traced back to impaired stabilization of the tricistronic *petL–petG–psaJ* RNA precursor and putative defects in the translation of *petL* and presumably a member of the *ndh* transcripts [66]. Further *in vitro* studies revealed an association of recombinant PGR3 with the 5′-UTRs of *petL* and *ndhA*, arguing for a role of PGR3 in the stabilization and translational activation of these two RNA targets [67]. Finally, analysis of several alleles harboring point mutations in different PPR motifs could decipher which parts of PGR3 are responsible for the RNA-binding activity and the translational activation of *petL* and *ndhA* [68]. Comparing the phenotypes of maize and *Arabidopsis pgr3* mutants revealed that the defects in *petL* stabilization and translation are conserved. However, as *pgr3* mutants in maize showed more dramatic effects than the *Arabidopsis* ones, it was concluded that ZmPGR3 has acquired additional functions compared with the *Arabidopsis* ortholog [69].

#### PPR5 stabilizes the *trnG*–UCC RNA precursor

The stabilizing function of PPR proteins is not restricted to protein coding mRNAs and rRNAs but extends to tRNAs, as demonstrated for the maize PPR5 protein that associates with the *trnG*–UCC precursor [70]. *In vitro* binding assays with recombinant ZmPPR5 could narrow down its binding site to the central region of the *trnG*–UCC intron, suggesting that PPR5 protects an endonuclease-sensitive site through its binding and might even facilitate the splicing of this group II intron [61] (Figure 1).

#### PPR103 stabilizes the 5′ end of *rpl16* in maize chloroplasts

Reverse-genetic approaches led to the identification of several PPR mutants exhibiting an embryo-defective phenotype indicating that they are indispensable for early plant development [71]. One of the identified mutants, *emb175*, exhibited a morphological arrest in early embryo development, pointing to a general house-keeping function of the respective PPR protein [72]. Analysis of the corresponding *ppr103* mutant in maize unveiled that the PPR103 protein stabilizes the processed *rpl16* mRNA by specific binding to its 5′-UTR [73]. Another interesting feature of PPR103 is its membership to the class of PLS-type proteins (PLS-class proteins contain alternating canonical P-type motifs and variant long (L)- and short (S)-type motifs) that are typically involved in RNA editing; however, a role of PPR103 in editing could be ruled out [73].

#### ATP4, a multifunctional PPR protein, involved in translation and stabilization of several chloroplast RNAs

Analysis of *Arabidopsis suppressor of variegation7* (*svr7*) mutant showed that rRNA processing and accumulation are affected causing a prevalent reduction of plastid proteins [74]. A later study of the maize ortholog ATP4 unveiled a function of ATP4 in promoting *atpA* and *atpB* translation [75]. Additionally, it was hypothesized that ATP4 plays a role in stabilizing the 3′ end of the *atpF–atpA* and *psaJ–rpl33* intergenic regions, thus acting on similar RNA targets as PPR10. Differences in the mutant phenotype in monocot and dicot species argue for evolutionary diverged functions of ATP4 and SVR7 [76].

#### CRR2 is required for the accumulation of processed *ndhB* transcripts

The *rps12–rps7–ndhB* precursor RNA is likely to be processed at not less than two sites in the *rps7–ndhB* intercistronic region. RNase protection assays revealed that the PPR protein CRR2 is required for intergenic processing of the site close to *ndhB* [77]. Additionally, CRR2 could stabilize the 5′ end of processed *ndhB* transcripts by binding to this region, thus hindering 5′-3′ exonucleolytic cleavage. This is reflected by the lack of 5′ processed *ndhB* transcripts whereas the precursor RNA accumulates at normal levels in *crr2* mutants in *Arabidopsis*, which are unable to accumulate stable and functional NADH dehydrogenase-like complexes mediating cyclic electron flow around photosystem I [77]. A further role in translation could not be excluded. In accordance with the proposed function of CRR2, its essential and C-terminal tripeptide motif DYW, consisting of aspartate (D), tyrosine (Y) and tryptophan (W), has been shown to be capable of cleaving RNA endonucleolytically *in vitro* even if the domain was considered to be related to RNA editing [78]. Interestingly, although there are no obvious significant differences between the DYW motifs, characteristic for many PPR proteins, the CRR2 DYW domain could not be replaced functionally by the apparently equivalent motifs of other PPR proteins. Thus, CRR2 is a DYW-dependent endoribonuclease with N-terminal PPR repeats likely to confer sequence-specificity to the protein [78]. Although binding of CRR2 to the *ndhB* 5′-UTR has never been investigated, its potential binding site is presumably represented by an abundant RNA footprint downstream of the 5′ end of the monocistronic *ndhB* mRNA implying that CRR2 could also function in the stabilization of processed RNAs; however, future research is needed to confirm this interaction [6,50].

#### PpPPR_38 is involved in splicing and stabilization of the *clpP* mRNA

The PPR531-11 protein from *Physcomitrella patens*, later renamed to PpPPR_38, was initially shown to be involved in splicing of *clpP* and processing of the *clpP–rps12* intergenic region resulting in elevated levels of precursor mRNAs [79]. In a following study, it was demonstrated that recombinant PpPPR_38 binds to the first intron of the *clpP* mRNA and to the *clpP–rps12* intergenic region *in vitro*. Studies with isolated chloroplast lysates suggested that binding of PpPPR_38 to the *clpP–rps12* intergenic region stabilizes a predicted stem–loop structure that first provides a correct intergenic endonucleolytic processing site and then protects the cleaved *clpP* mRNA against 3′→5′ exoribonuclease activity [80].

### CpRNPs are general RNA stabilization factors in chloroplasts

In general cpRNPs are divided into three different groups according to predicted structural features and phylogenetic considerations: I (cp28 and cp31), II (cp29A and cp29B), and III (cp33) [81]. CpRNPs are RNA-Recognition Motifs (RRM)-containing, abundant stroma-localized proteins that associate with various ribosome-free mRNAs and pre-tRNAs [82,83]. Interestingly, depletion of the cpRNPs in stromal extracts was followed by rapid RNA degradation while addition of recombinant cpRNPs could restore RNA-stability, suggesting that cpRNPs act as general mediators for RNA stabilization [84]. Meanwhile, in-depth analyses of some of the cpRNP members are available, confirming their involvement in post-transcriptional processes. Spinach 28RNP was suggested to be required for processing and stabilization of several 3′ ends of chloroplast mRNAs including *psbA, rbcL, petD*, and *rps14* as revealed by *in vitro* studies [85]. In addition, recombinant 28RNP was assumed to be regulated via phosphorylation as its RNA-binding affinity decreased 3- to 4-fold upon phosphorylation [86]. In contrast, 24RNP exhibits an opposite mode of regulation, as phosphorylation enhances the affinity toward its targets [87]. CP31A in *Arabidopsis* has a more specific function and was shown to be important for editing of different sites and stabilization of several but mainly *ndhF* mRNAs, whereas CP31B was assumed to support editing of specific CP31A-dependent sites [88]. Further transcriptome-wide analysis revealed that two cpRNPs with overlapping function, CP31A and CP29A, associate with the 3′ end of numerous sense and antisense transcripts including *psbB, psbD, psaA/B, atpB*, and *ndhB* to protect them from degradation under cold stress conditions [89]. The *Arabidopsis* CP33A protein is another example for the global role of cpRNPs in processing and stabilizing of multiple chloroplast RNAs [90].

### Additional chloroplast RNA-stabilizing RNA-binding proteins

#### HCF145 stabilizes the polycistronic *psaA–psaB–rps14* mRNA at the 5′ end

The seedling-lethal *hcf145* mutant was initially found to specifically lack PSI [91]. This coincided with a severe destabilization of the tricistronic *psaA–psaB–rps14* transcript encoding the two photosystem I core proteins PsaA and PsaB. Binding of HCF145 to the *psaA* 5′-UTR probably protects it from exoribonucleotytic attack [92]. The HCF145 protein contains two highly homologous transcript-binding motif repeat (TMR) domains of approximately 70 amino acids which are responsible for specific binding to the *psaA* 5′-UTR. The TMR motifs are present rarely as single domains but often as multiple (up to ten) repeats in quite diverse proteins of unknown function in photosynthetic organisms including moss, red and green algae, and cyanobacteria [92]. The predicted helical TMR motifs probably add a new example to the repertoire of RNA-binding domains in photosynthetic organisms.

Two additional N-terminal repeated regions of unknown function were found in the HCF145 protein that showed homology to the ubiquitously distributed SRPBCC (START/RHO_alpha_C/PITP/Bet_v1/CoxG/CalC) binding domains superfamily consisting of 11 Pfam families, including START domains, phosphatidylinositol transfer proteins, Bet_v_1, CalC related, CoxG, and polyketide cyclase related families [93]. The structural similarity of the large ligand binding domains to already crystallized members of known function and ligands suggests that they either bind hydrophobic ligands like lipids, phytohormones, steroids, coenzyme Q, alkaloids, polyketides, and/or represent a catalytic pocket for the production of complex polyaromatic substances of the secondary metabolism, such as aromatic hydrocarbon hydroxylating enzymes. Structurally related SRPBCC domains are present in 37 *Arabidopsis* proteins with unknown function and localization [92].

Interestingly, the SRPBCC-related ligand-binding motifs support association to the 5′ *psaA* mRNA, although they do not contribute to the binding on their own. This points to a regulatory role of the SRPBCC domains in adjusting PSI levels [92] and provides a control of metabolic processes in fine-tuning RNA binding and thus stabilization of the *psaA* RNA. Further studies will show whether a metabolism-dependent regulation of *psaA–psaB–rps14* mRNA levels is important during development, greening and/or acclimation processes.

#### PrfB1 is required for stabilization of UGA stop codon-containing transcripts

Different from eukaryotes harboring only one release factor (eRF) for ribosomal release at all three stop codons, eubacteria and organelles contain two release factors, PrfA (RF1) and PrfB (RF2), for termination of translation at UAG and UGA stop codons respectively. The UAA codon is recognized by both factors. PrfB1 and PrfB2 encode the only functional plastid and mitochondrial ribosomal RF2-related proteins in land plants respectively [94]. In contrast with numerous eubacterial *prfB* mutants described so far, in *Arabidopsis* lines lacking PrfB1 most plastid UGA stop codon-containing transcripts are unstable [94,95]. The essential PrfB1 protein typically harbors two most important tripeptide motifs characteristic for all organellar and eubacterial PrfB homologs: the “SPF” stops codon recognition motif and the “GGQ” reaction center for peptidyl-tRNA hydrolysis. In some algae, such as *C. reinhardtii*, a PrfB-related gene is lacking in the nuclear genome and accordingly TGA stop codons are absent in their plastid genomes [94]. It is likely that similar to eubacterial systems lack of mutations of PrfB causes frame shifting at UGA stop codons which produces altered C-termini of different length depending on the position of the next in-frame UAG or UAA stop codons. Either these undesirable reading mistakes or read-through into the 3′-UTR are responsible for destabilization of plastid transcripts harboring UGA stop codons in *prfB1* mutants. In either case, a hitherto unrecognized surveillance system efficiently removes all these nonfunctional transcripts. This demonstrates an advanced and more sophisticated regulatory level of plastid gene expression in comparison with known eubacterial systems.

Interestingly, a possible regulatory function of translational termination at UGA stop codons is reflected by a significant increase in the TGA content in land plant plastid genomes in comparison with those in algae [94]. Presumably, evolutionary constraints keep the number of plastid TGA stop codon high in land plants in order to deal with tissue specificity, developmental programs, and/or changing environmental conditions during acclimation processes on the molecular level.

#### PrfB3 stabilizes the *petB* mRNAs with cleaved 3′ ends

Land plant genomes surprisingly encode an additional eubacterial RF2-related protein, named PrfB3, which lost its two most important “SPF” and “GGQ” motifs of a release factor and thus the capability to terminate translation at UGA and UAA stop codons [31]. Accordingly, PrfB3 mutants in *Arabidopsis* are able to terminate translation at UGA stop codons but are specifically affected in the accumulation of the cytochrome *b*_6_*f* complex due to the lack of *petB* transcripts encoding cytochrome *b*_6_. The *petB* gene is part of the primary pentacistronic *psbB–psbT–psbH–petB–petD* transcript encoding three proteins of PSII (PsbB, PsbT, and PsbH) and two proteins of the cytochrome *b*_6_*f* complex (PetB and PetD). Similar to most plastid polycistronic transcripts, numerous endonucleolytic processing events generate a wealth of overlapping RNA species, which are subjected to individual life times and translational control. Data revealed that *petB* transcripts that are cleaved at the 3′ end are efficiently generated but rapidly degraded in *prfB3* mutants indicating that PrfB3 protects these RNAs from 3′→5′ exonucleolytic attacks. This is in agreement with preferential binding of PrfB3 to the *petB* 3′-UTR with respect to control transcripts [31]. Surprisingly, spliced but 3′ unprocessed *petB*-containing precursor transcripts accumulate in *prfB3* but *petB* is not translated. This indicates that binding of PrfB3 to the 3′ end could have an impact on elongation and/or initiation of translation at the 5′ end or that only processed monocistronic *petB* transcript are translational competent. Alternatively, a potential weak binding of PrfB3 also to the *petB* 5′-UTR is required for efficient initiation of translation [31].

A drop in the expression of PrfB3 in partially complemented mutant lines correlated with a decrease in *petB* mRNA levels [31]. Most importantly, a remarkable decrease in the accumulation of PrfB3 upon changing light conditions and in photoreceptor mutants was also accompanied to a certain extent by a loss of cytochrome *b*_6_*f* amounts. Therefore, PrfB3 could well serve a regulatory function *in vivo* during acclimation processes by rate limiting levels of *petB* transcripts when changes in the amount of the cytochrome *b*_6_*f* complex are required. A similar regulatory role was assigned to the plastid PPR protein MCA1 in Chlamydomonas in determining levels of *petA* mRNA and accordingly its product cytochrome *f* [96].

#### HCF107 is required for accumulation of the *psbH* mRNA

The HCF107 gene was initially identified by high resolution mapping of the corresponding *Arabidopsis* mutation, which was characterized by a lack of some but not all oligo- and monocistronic *psbH* transcripts generated by processing of the pentacistronic *psbB–psbT–psbH–petB–petD* precursor message [55,56]. Determination of transcript termini demonstrated that only transcripts that are processed at position -45 in the 5′-UTR are lacking in the mutant [56]. All other transcripts of this operon accumulate at normal levels and size already indicating that stability of the processed *psbH* messages rather than processing is affected. *PsbH* encodes an essential 8 kDa phosphoprotein of PSII whose absence leads to a pronounced deficiency of PSII activity and protein levels in the *hcf107* mutant [55,56]. Ectopic expression of *psbH* in the nuclear genome, which targets the protein back to the chloroplast, revealed that HCF107 is exclusively required for the expression of *psbH* [97]. This is in contrast with the function of the evolutionary ortholog MBB1 in *C. reinhardtii* that affects expression of *psbB* [37].

HCF107 encodes a half-a-tetratricopeptide (HAT) protein containing 11 repeats and no additional domains [98]. It is mostly associated with thylakoid membranes and forms high molecular weight complexes of 60–190 and 600–800 kDa [29]. Crystal structure analysis of HAT proteins revealed that they form helical α-solenoid hairpins similar to TPR proteins and that they are capable of binding RNA. Accordingly, HAT proteins are involved in splicing, polyadenylation, and the maturation of ribosomal RNAs [99]. It appeared that HCF107 binds specifically 11 nucleotides in the 5′ leader 44 nucleotides upstream of the *psbH* start codon in agreement with the one repeat—one nucleotide model of PPR proteins [98] and according to the 5′ mapped *psbH* transcripts lacking in *hcf107*. Expectedly, binding to its target protects the *psbH* RNA from 5′→3′ exonucleolytic attack *in vitro* [98]. RNA structure probing revealed that binding of HCF107 to the *psbH* 5′-UTR remodels the RNA structure, which may also allow ribosome binding and subsequent enhancement of translation, hinting to a dual role of the HCF107 protein. Obviously, PPRs and HATs can act in a similar and presumably analogous way to the recently identified family containing predicted superhelical and repeated RNA-binding TMR motifs in photosynthetic organisms [92].

## Conclusion and outlook

Here, we summarized the characteristics of plastid RNA-binding proteins with a protective role in vascular plants. Whether the regulation of endonucleolytic activities, which were assumed to initiate degradation, is rate-limiting for mRNA decay still remains elusive. The RNA-binding proteins addressed here evolved mostly to hinder exonucleolytic attacks of tRNAs and translationally competent mRNAs, some of which were also shown to impact additional processing events and/or translation. Ribosome profiling was shown to partially overcome the challenge to investigate the role of the protectors in translation in particular using genetic approaches with low abundant RNA targets in the respective mutants [63,100]. Nevertheless, questions need to be addressed regarding a direct involvement of the RNA protectors in translational stimulation, e.g. by recruiting additional proteins or restructuring the mRNA to render it accessible for ribosomes. Thus, chloroplast RNA-binding protectors could also act as RNA chaperones, which resolve kinetically trapped misfolded RNA structures to produce functional RNAs, e.g. translationally competent RNAs, which are accessible for ribosomes. In addition, the function of the recently identified chloroplast noncoding RNAs in the overall context of RNA stability still remains to be determined [6,58]. Furthermore, the relationship between mRNA abundance, translational rates of individual mRNA species, and accumulation of the respective gene product in correlation to RNA protectors needs to be investigated quantitatively and under changing environmental conditions to identify rate-limiting steps in plastid gene expression.

Differential expression of RNA protector genes under changing environmental conditions and the effects on their downstream targets speaks in favor of the idea that RNA degradation is mostly regulated at the RNA extremities. One example is provided by a comparable differential expression of AtprfB3 and its target *petB* in *Arabidopsis* [31]. Whether PrfB3 is indeed rate limiting for the amounts of the cytochrome *b*_6_*f* complex during stress and/or acclimation processes still needs to be shown.

The progression in mass spectrometric methods will prompt future studies on the metabolic impact and post-translational modifications of RNA protectors on the regulation of their activity, interaction with other hitherto unidentified association partners, and/or RNA affinity in a quantitative manner. Generally, these analyses remained poor, nevertheless the potential integrator HCF145 seems to be a promising candidate for such experiments. The two SRPBCC ligand binding domains of HCF145 offer an excellent platform for their metabolic control by the integration of external stimuli to finally fine-tune photosynthesis by regulating the effect of HCF145 on *psaA* stability and thus photosystem I amounts [91]. Also, cpRNPs deserve a closer look as they were reported to be of particular importance under cold stress conditions and influence a wide range of target transcripts [88]. Studies on chloroplast gene expression is important not only for understanding regulatory aspects but also for guiding transplastomic approaches for scientific and biotechnological purposes in model as well as agriculturally important plants [3,4].

Most protectors are plant specific congruent with the fast divergence of their targets at the 5′- and 3′-UTRs of plastid transcripts [101]. Even if counterparts of the RNA-binding proteins are occasionally found in algae or even rarely in cyanobacteria, they exert a different function on transcripts or have different targets. This demonstrates that the RNA metabolism represents a fast-evolving process, which presumably reflects a combined adaptation to multicellularity, developmental programs, and changes in light, temperature, humidity, and water supply. Specificity factors, such as plastid RNA protectors, may integrate external signals on the molecular level to rapidly cope with changes of environmental conditions allowing to perform stress responses and acclimation processes. This is reflected by changes in plastid RNA patterns and abundance during development and greening, in different tissues and under changing environmental conditions [9].

## Summary

- Chloroplast gene expression is predominantly governed on the level of post-transcriptional processes including RNA editing, methylation, polyadenylation, splicing, endo- and exonucleolytic digestion as well as translation.
- Generally, newly evolved and vascular plant-specific RNA-binding factors encoded in the nuclear genome are required for protecting specific chloroplast RNAs at exo- and endoribonuclease-sensitive sites reflecting a fast-evolving RNA metabolism congruent with the diverging RNA targets.
- Chloroplast RNA-binding protectors, usually found in high molecular weight complexes with largely unknown components, can act as RNA chaperones, occasionally affect other post-transcriptional processes, such as splicing, editing and translation, and contain a variety of different RNA recognition motifs, which often appear as multiple repeats.
- Interactions with other proteins, post-translational modifications of protectors, metabolic pathways, and presumably noncoding RNAs may have an important impact on the characteristics of RNA protectors, which are required for normal plant growth and development as well as diverse stress responses and acclimation processes.

## Abbreviations

cpRNP: chloroplast ribonucleoprotein
HAT: half-a-tetratricopeptide repeat
NEP: nuclear-encoded RNA polymerase
OPR: octatricopeptide repeat
PEP: plastid-encoded bacterial type RNA polymerase
PPR: pentatricopeptide repeat
RRM: RNA-recognition motifs
TMR: transcript-binding motif repeat
